# Identification of low and very high-risk patients with non-WNT/non-SHH medulloblastoma by improved clinico-molecular stratification of the HIT2000 and I-HIT-MED cohorts

**DOI:** 10.1007/s00401-022-02522-4

**Published:** 2022-12-02

**Authors:** Martin Mynarek, Denise Obrecht, Martin Sill, Dominik Sturm, Katja Kloth-Stachnau, Florian Selt, Jonas Ecker, Katja von Hoff, Björn-Ole Juhnke, Tobias Goschzik, Torsten Pietsch, Michael Bockmayr, Marcel Kool, Andreas von Deimling, Olaf Witt, Ulrich Schüller, Martin Benesch, Nicolas U. Gerber, Felix Sahm, David T. W. Jones, Andrey Korshunov, Stefan M. Pfister, Stefan Rutkowski, Till Milde

**Affiliations:** 1 Department for Pediatric Hematology and Oncology, University Medical Center Hamburg-Eppendorf, Martinistr. 52, 20246 Hamburg, Germany; 2 Mildred Scheel Cancer Career Center HaTriCS4, University Medical Center Hamburg-Eppendorf, Hamburg, Germany; 3 Hopp Children’s Cancer Center Heidelberg (KiTZ), Im Neuenheimer Feld 280, 69120 Heidelberg, Germany; 4 Division of Pediatric Neurooncology, German Cancer Research Center (DKFZ) and German Consortium for Translational Cancer Research (DKTK), Heidelberg, Germany; 5 Department of Pediatric Hematology and Oncology, Heidelberg University Hospital, Heidelberg, Germany; 6 Division of Pediatric Glioma Research, German Cancer Research Center (DKFZ) and German Consortium for Translational Cancer Research (DKTK), Heidelberg, Germany; 7 Clinical Cooperation Unit Pediatric Oncology, German Cancer Research Center (DKFZ) and German Consortium for Translational Cancer Research (DKTK), Heidelberg, Germany; 8 Charité-Universitätsmedizin Berlin, Berlin, Germany; 9 Institute of Neuropathology, Brain Tumor Reference Center of the German Society for Neuropathology and Neuroanatomy (DGNN), University of Bonn Medical Center, Bonn, Germany; 10 Research Institute Children’s Cancer Center Hamburg, Hamburg, Germany; 11 Princess Máxima Center for Pediatric Oncology, Utrecht, The Netherlands; 12 Department of Neuropathology, Institute of Pathology, University Hospital Heidelberg, Heidelberg, Germany; 13 Clinical Cooperation Unit Neuropathology, German Consortium for Translational Cancer Research (DKTK), German Cancer Research Center (DKFZ), Heidelberg, Germany; 14 Department of Neuropathology, University Medical Center Hamburg-Eppendorf, Hamburg, Germany; 15 Division of Pediatric Hematology and Oncology, Department of Pediatrics and Adolescent Medicine, Medical University of Graz, Graz, Austria; 16 Department of Oncology, University Children’s Hospital, Zurich, Switzerland; 17 Children’s Research Centre, University Children’s Hospital, Zurich, Switzerland

**Keywords:** Medulloblastoma, Non-WNT/non-SHH, Group 3/4, Risk stratification

## Abstract

Molecular groups of medulloblastoma (MB) are well established. Novel risk stratification parameters include Group 3/4 (non-WNT/non-SHH) methylation subgroups I–VIII or whole-chromosomal aberration (WCA) phenotypes. This study investigates the integration of clinical and molecular parameters to improve risk stratification of non-WNT/non-SHH MB. Non-WNT/non-SHH MB from the HIT2000 study and the HIT-MED registries were selected based on availability of DNA-methylation profiling data. *MYC* or *MYCN* amplification and WCA of chromosomes 7, 8, and 11 were inferred from methylation array-based copy number profiles. In total, 403 non-WNT/non-SHH MB were identified, 346/403 (86%) had a methylation class family Group 3/4 methylation score (classifier v11b6) ≥ 0.9, and 294/346 (73%) were included in the risk stratification modeling based on Group 3 or 4 score (v11b6) ≥ 0.8 and subgroup I–VIII score (mb_g34) ≥ 0.8. Group 3 MB (5y-PFS, survival estimation ± standard deviation: 41.4 ± 4.6%; 5y-OS: 48.8 ± 5.0%) showed poorer survival compared to Group 4 (5y-PFS: 68.2 ± 3.7%; 5y-OS: 84.8 ± 2.8%). Subgroups II (5y-PFS: 27.6 ± 8.2%) and III (5y-PFS: 37.5 ± 7.9%) showed the poorest and subgroup VI (5y-PFS: 76.6 ± 7.9%), VII (5y-PFS: 75.9 ± 7.2%), and VIII (5y-PFS: 66.6 ± 5.8%) the best survival. Multivariate analysis revealed subgroup in combination with WCA phenotype to best predict risk of progression and death. The integration of clinical (age, M and R status) and molecular (MYC/N, subgroup, WCA phenotype) variables identified a low-risk stratum with a 5y-PFS of 94 ± 5.7 and a very high-risk stratum with a 5y-PFS of 29 ± 6.1%. Validation in an international MB cohort confirmed the combined stratification scheme with 82.1 ± 6.0% 5y-PFS in the low and 47.5 ± 4.1% in very high-risk groups, and outperformed the clinical model. These newly identified clinico-molecular low-risk and very high-risk strata, accounting for 6%, and 21% of non-WNT/non-SHH MB patients, respectively, may improve future treatment stratification.

**Supplementary Information:**

The online version contains supplementary material available at 10.1007/s00401-022-02522-4.

## Introduction

Risk stratification for medulloblastoma (MB) patients remains a challenge, and recent advances in molecular understanding of the disease spectrum have provided novel risk markers. In this manuscript, we will refer to the molecular MB groups WNT, SHH, Group 3 and 4 as “groups”, and to the subgroups or -types (e.g. Group 3/4 subgroups I–VIII) as “subgroups”, to account for the most recent changes in the WHO classification of tumors of the central nervous system (CNS) [28]. Epigenetic groups (WNT, SHH, Group 3 and 4) of MB have been well described for over a decade [26], and more recently, subgroups within the groups have been identified [4, 20, 24]. While two potential subgroups have been described in the WNT group [4], the SHH group segregates in four subgroups [4, 10, 18, 22] with distinct age- and risk profile, and recognition of the four subgroups SHH-1, SHH-2, SHH-3, SHH-4 in the current WHO CNS5 classification. For the classification of non-WNT/non-SHH MB, data from Kool et al. indicated early on a significant overlap [12]. Indeed, the segregation into the two groups Group 3 and Group 4 is now recognized as being not fully discriminating, and recent data demonstrate segregation of non-WNT/non-SHH MB into eight distinct subgroups (I–VIII) [20, 24]. The current 2021 update CNS5 of the WHO classification of tumors of the CNS [14] recognizes DNA methylation-based classification as a diagnostic method and describes the aforementioned eight subgroups within non-WNT/non-SHH MB. Associations with prognosis have been suggested for the subgroups, but the specific clinical implications remain to be studied prospectively.

A retrospective analysis of MB groups [23] identified low-risk (5-year progression-free survival [5y-PFS] 91%) (WNT; SHH without risk factors; Group 3/4 (G3/4) with chr13 loss and no *MYC* amp), average or standard risk (5y-PFS 81%) (G3/4 low-risk without *MYC* amp), high-risk (5y-PFS 42%) (G3/4 HR without *MYC* amp), and very high-risk strata (5y-PFS 28%) (SHH with R + or M + or large cell anaplastic (LCA) or *MYCN* amp; G3/4 with *MYC* amp), indicating clinical variability within MB groups, including non-WNT/non-SHH MB. However, this study was performed before the description of the eight G3/4 subgroups. In the clinical trial setting, MB subgroups have been analyzed retrospectively most recently in the SJMB03 trial [7]. Combining clinical criteria with MB subgroups, Gajjar et al. identified a low-risk stratum within the G3/4 MB, defined by M0 and subgroup VII [7].

Besides methylation-based classification, the number of whole-chromosomal aberrations (WCA) has been suggested as another molecular stratification scheme for non-WNT/non-SHH MB. Shih et al. described chromosome 11 loss and chromosome 17 gain as a marker for favorable prognosis in non-metastatic Group 4 MB [25]. Goschzik et al. identified a low-risk subgroup within the non-WNT/non-SHH MB defined by gains and losses of whole arm chromosomal aberrations of chromosome 7, 8, and 11, within the PNET4 cohort and an independent retrospective validation cohort, with a 5y-PFS of 100% and 95%, respectively [9]. Validation of the WCA signature is important to the field, since the original study of Goschzik et al. is the only study to report its derivation to date.

To integrate clinical and molecular information we analyzed the HIT2000 and I-HIT-MED cohorts with the goals to analyze the prognostic value of MB subgroups I–VIII and further optimize the risk stratification for non-WNT/non-SHH MB, and provide validation in a separate MB cohort.

## Materials and methods

### Study type and patient cohorts

**Fig. 1:**
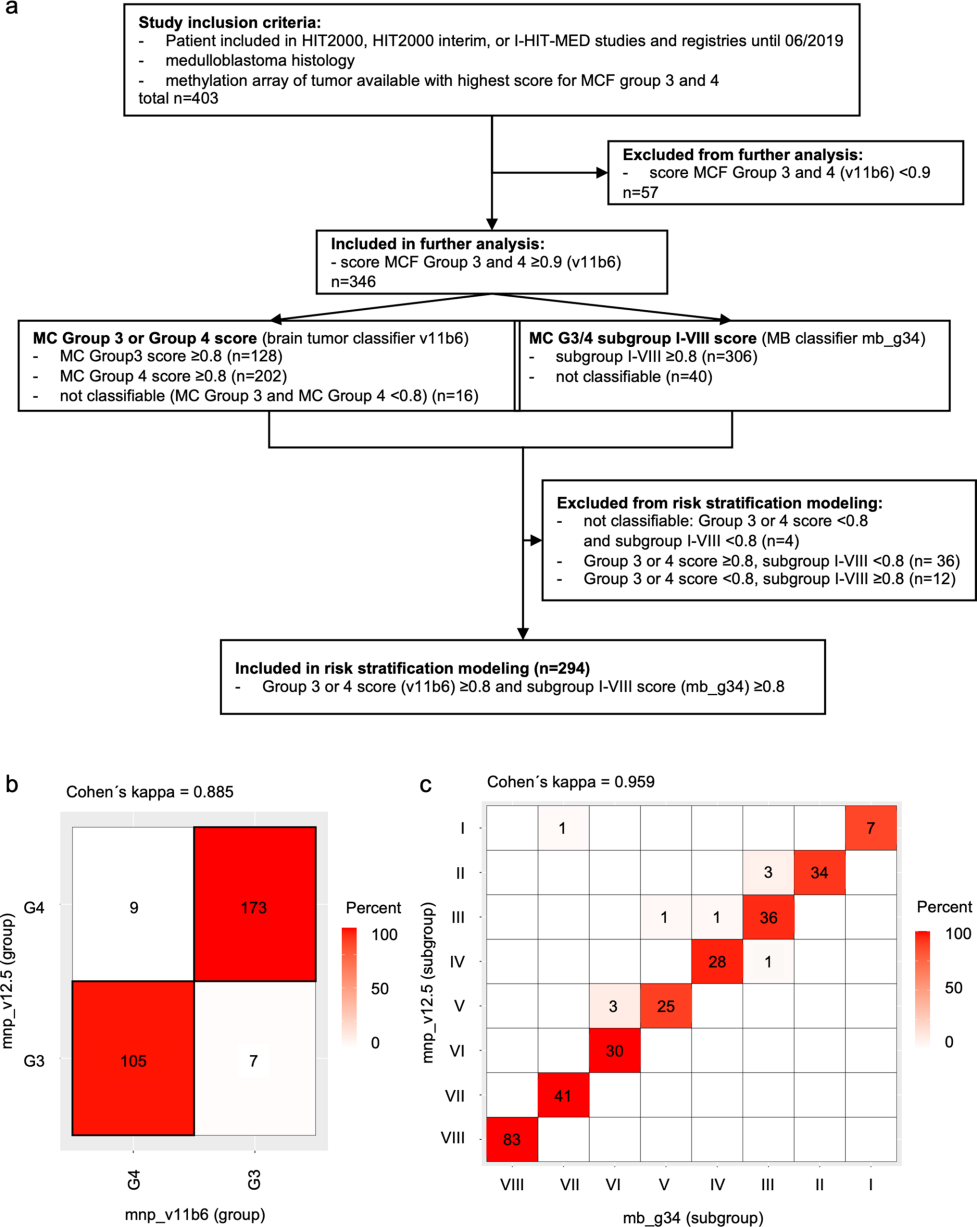
Study cohort and methylation classification. **a** Consort diagram of the HIT study cohort. *MC* methylation class, *MCF* methylation class family, *MB* medulloblastoma. **b** Agreement between group 3 and 4 predictions of the v11b6 (mnp_v11b6) and the v12.5 (mnp_v12.5) classifier. The predicted groups differ for only 16 out of 294 samples, indicating a high inter-observer reliability between both classifiers with an almost perfect Cohen’s kappa of 0.885. **c** Agreement between subgroup predictions of the v12.5 (mnp_v12.5) and the MB group 3 and 4 (mb_g34) classifier. The predicted subgroups differ for only ten out of 294 samples, which indicates a high inter-observer reliability between both classifiers with an almost perfect Cohen's kappa of 0.959

This is an international, retrospective, multi-center study. Patients were eligible, if they had (a) histologically confirmed MB, (b) DNA-methylation profiling that was classified to belong to the methylation class family (MCF) Group 3/4 by the Heidelberg Brain Tumor classifier v11b6 with a score of ≥ 0.9, (c) had their first tumor surgery before June 2019 and (d) participated in one of the following trials or registries: the HIT2000 trial (NCT00303810), HIT2000interim registry (NCT02238899) or I-HIT-MED registry (NCT02417324) (Fig. 1a). The HIT2000 trial and the HIT2000interim registry were approved by the ethic committee of the Medical Faculty, University Würzburg, the I-HIT-MED registry was approved by the ethic committee of the Medical Faculty, University Hamburg. All cases were subject to central review of MR imaging, histopathology, and CSF. All patients or their legal representatives gave their written informed consent into participation before inclusion into the respective project. Patients were allocated to the Sharma cohort (*n* = 163), included in a previous subgroup meta-analysis [24], or the extension cohort (*n* = 183) (not previously published). Only 4/294 (1.4%) cases overall and 4/56 (7.1%) standard risk cases are included in both the HIT as well as the published PNET cohort [9], allowing for validation of the WCA phenotypes identified by Goschzik et al. in our separate HIT cohort.

Therapy was given either according to the HIT2000 trial protocol (participant of the HIT2000 trial) or according to institutional guidelines (registry patients). Patients younger than four years at first tumor surgery received radiation-sparing chemotherapy with intraventricular methotrexate and HIT-SKK chemotherapy for non-metastatic disease and modified Head-Start chemotherapy followed by high-dose chemotherapy in patients with metastatic disease. Craniospinal irradiation (CSI) was reserved for patients with incomplete response or relapse. Patients older than four years received PNET4-like therapy [13], if no high-risk characteristics were present, or MET-HIT2000-AB4-like therapy in case of metastatic disease [27], including CSI. Adherence to a specific protocol was not prerequisite for inclusion into this analysis. For non-trial participants, classification on “infant-type” vs. “primary CSI-containing” therapy strategy was done based on documented therapy courses since information on intended therapy protocol was not available.

### Methylation profiling and sample classification

DNA-methylation profiles were generated through the molecular diagnostic studies PTT2.0 (Ecker J et al., submitted; German Clinical Trial Register DRKS00011707; https://www.kitz-heidelberg.de/en/clinical-studies/molecular-diagnostics-studies/ptt-20/) and MNP2.0 (Sturm D et al., submitted; https://www.kitz-heidelberg.de/en/clinical-studies/molecular-diagnostics-studies/mnp-20/), or in the context of retro- and prospective analyses of the HIT2000 trial cohort [21, 27]. Genomic DNA was extracted from fresh-frozen or formalin-fixed and paraffin-embedded (FFPE) tissue samples. DNA-methylation profiling of all samples was performed using the Infinium MethylationEPIC (850 k) BeadChip (Illumina, San Diego, CA, USA) or Infinium HumanMethylation450 (450 k) BeadChip array (Illumina) as previously described [3]. Data of the samples of Sharma, 2019 [24] and Cavalli, 2017 [4], were retrieved from Gene Expression Omnibus (GEO, https://www.ncbi.nlm.nih.gov/geo/) with the identifiers GSE130051 (Sharma) and GSE85218 (Cavalli), respectively. All computational analyses were performed in R version 3.5.3 (R Development Core Team, 2022; https://www.R-project.org). Raw signal intensities were obtained from IDAT-files using the minfi Bioconductor package version 1.21.4 [1]. Illumina EPIC samples and 450 k samples were merged to a combined data set by selecting the intersection of probes present on both arrays (combineArrays function, minfi). Each sample was individually normalized by performing a background correction (shifting of the 5% percentile of negative control probe intensities to 0) and a dye-bias correction (scaling of the mean of normalization control probe intensities to 10,000) for both color channels. Subsequently, a correction for the type of material tissue (FFPE/frozen) and array type (450 k/EPIC) was performed by fitting univariable, linear models to the log2-transformed intensity values (removeBatchEffect function, limma package version 3.30.11). The methylated and unmethylated signals were corrected individually. Beta-values were calculated from the retransformed intensities using an offset of 100 (as recommended by Illumina). All samples were checked for duplicates by pairwise correlation of the SNP probes on the 450 k/850 k array. Filtering of CpG probes was performed as described in Capper et al. 2018 [3]. In total, 428,230 probes were kept for downstream analysis. To perform unsupervised non-linear dimension reduction, the remaining probes after standard filtering were used to calculate the 1-variance weighted Pearson correlation between samples. The resulting distance matrix was used as input for t-SNE analysis (t-Distributed Stochastic Neighbor Embedding; Rtsne package version 0.13). The following non-default parameters were applied: theta = 0, pca = F, max_iter = 2,500 perplexity = 30. Copy number variation (CNV) analysis from 450 k and EPIC methylation array data was performed using the conumee Bioconductor package version 1.12.0. Recurrent aberrations and total losses and gains of chromosomal arms were called by visual inspection of copy number profile plots (see below).

Tumor classification of methylation class family (MCF) and methylation class (MC) Group 3 and 4 was performed by applying the Heidelberg DNA methylation-based classification tool [3, 15] (v11b6). For subgrouping of Group 3 and Group 4 MBs into the consensus methylation classes subgroups I-VIII presented in Sharma et al. 2019 [24] a novel similar classification tool (medulloblastoma classifier group 3/4: MB_g34) was used. Both classifiers are publicly available at www.molecularneuropathology.org. A calibrated score threshold of ≥ 0.9 For MCF Group 3 and 4, and of ≥ 0.8 for MC Group 3 or 4, was applied to consider a class prediction reliable with the brain tumor classifier v11b6 [3, 15]. For classification of MC Group 3/4 subgroups I–VIII [24] a score of ≥ 0.8 (MB_g34) was accepted with the MB_g34 classifier. This multi-step approach ensured that all samples included were definitely Group 3/4 MB by meeting stringent criteria for the MCF Group 3 and 4 (v11b6 ≥ 0.9 for MCF Group 3 and 4), but did not unnecessarily reduce sample size due to sample exclusion using an acceptable threshold of ≥ 0.8 for MC Group 3 or 4 (v11b6), and subgroups I–VIII within Group3/4 (MB_g34).

### Manual MYC, MYCN amplification, isochromosome 17q and whole-chromosomal aberration calling

Methylation-array-based copy number plots [11] were used to manually call *MYC* or *MYCN* amplifications, chromosomal aberrations of chromosome 17p and 17q, and whole arm chromosomal aberrations of chromosome 7, 8, and 11. A threshold of ≥ 0.4 (log2 Copy number ratio) and clearly distinct from baseline was used to call amplifications of *MYC* or *MYCN*, and of ≥ 0.2 (log2 Copy number ratio) from baseline to call gains or losses of chromosome arms. FISH, Multiplex Ligation-dependent Probe Amplification (MLPA) or Molecular Inversion Profiling (MIP) data on *MYC*/*MYCN* amplifications were available for 130 of the samples from the HIT cohort. Comparison of *MYC*/*MYCN*-amplification detection by methylation profiling-derived copy number analysis and detection by FISH, MLPA or MIP showed a high concordance between methylation profiling-derived copy number analysis and the other methods (Cohen’s Kappa 0.88 [MYC] and 0.84 [MYCN]; see Supplemental Table 1, online resource). Based on the high concordance we continued our analyses using the information on MYC/MYCN amplification based on methylation profiling.

A threshold of ≥ 80% of the length of the respective chromosome arm was used to call gains or losses of a chromosome arm. An isochromosome 17q (i17q) was called, if both a loss of chromosome arm 17p as well as a gain of chromosome arm 17q was present. A whole-chromosomal aberration (WCA) of chromosome 7 (chr7 gain) was called, if both chromosome arms of chromosome 7 were gained, a WCA of either chromosome 8 (chr8 loss) or 11 (chr11 loss) was called, if both chromosome arms of either chromosome 8 or 11, respectively, were lost. A WCA favorable risk (FR) phenotype was called if ≥ 2 WCA (of chr7, 8 or 11) were present [9]. All three markers of the WCA phenotype were validated in the clinically standard risk patients of the HIT cohort (*n* = 56) (Supplemental Figs. 1 and 2, online resources). The best prediction of PFS was achieved by the combination of all three markers (Supplemental Fig. 1c, online resource).

### Validation cohort

The validation cohort consisted of non-WNT/non-SHH medulloblastoma selected from the international medulloblastoma series described previously [6, 24]. The validation cohort consisted of histologically confirmed MB, with DNA-methylation classification of Group 3 and 4, as well as subgroups I-VIII done as described above. Therapy was done according to various protocols as described [6]. *MYC* or *MYCN* amplifications and whole-chromosomal aberrations of chromosome 7, 8, and 11 were identified by CNV methylation plots. All *MYC*/*MYCN* amplifications called were validated by iFISH. Classification of “infant-type” vs. “primary CSI-containing” therapy was done as for the HIT cohort.

### Statistical analyses

This study aimed at investigating how integration of biological risk factors can improve prediction of risk of progression in non-WNT/non-SHH medulloblastoma. For this we generated multivariable Cox-regression models on PFS combining known clinical and molecular risk factors (Staging, use of craniospinal radiotherapy, MYC amplifications and MYCN-Amplifications) with different, risk factors currently not established in clinical routine (Group 3 vs. Group 4, WCA-classification, subgroups I–VIII). The model prediction was quantified by comparing the area under the curve (AUC) of a receiver operating characteristic (ROC)-curve for PFS five years after diagnosis using the R-package risksetROC. The optimal model to predict the outcome was selected by the combination of established risk factors with new molecular factors which had the highest AUC. Concordance index and Akaikes information criterion (AIC) were used additionally to describe the model quality.

This information was then used as a basis to select parameters for a clinically applicable model predicting progression-free survival in the dataset. Comparison of the new “clinico-molecular” model was subsequently compared versus the currently established “clinical” model by following the Brier score overt time (“prediction error curve”) and integrating the prediction error curves in an “integrated Brier Score” (IBS) as well as the relative C-indices. Both IBS and the concordance index were cross-validated based on bootstrap resampling in 500 boostrap-samples. Finally, the results were confirmed in the validation cohort, by predicting the survival probabilities applying models fitted to the complete discovery cohort and comparing the IBS for PFS up to 5 years after diagnosis and the C-index. [8, 17]

PFS was defined as time from first tumor surgery to progression, relapse or death from any cause. Overall survival (OS) was defined as time from first tumor surgery to death from any cause. Both were censored at last follow-up for patients without an event. Survival rates were estimated using the Kaplan–Meier method. Standard deviation was displayed for all estimations. Survival differences were analyzed using the log-rank test for univariable comparisons. Because radiotherapy (RT) is considered a very important predictor for survival in MB but its use may be very dependent on the protocol especially in younger children, use of CSI was modeled as a time-dependent covariate in Cox-regression analysis. Age was not included into the “known clinical and molecular risk factors” because it is strongly correlated with therapy selection and therefore use of CSI. Inferential statistics are intended to be exploratory (hypotheses generating), not confirmatory, and are interpreted accordingly. The comparison-wise type I error rate was controlled instead of the family-wise error rate. The local significance level was set to 0.05. No adjustment for multiple testing was performed. To test statistical independence of categorical variables the *χ*^2^ test was applied. All analyses were done with R Version 4.2.0 with packages specified above together with survival, survminer, cmprsk, and ggplot2.

## Results

### Cohort description

**Fig. 2:**
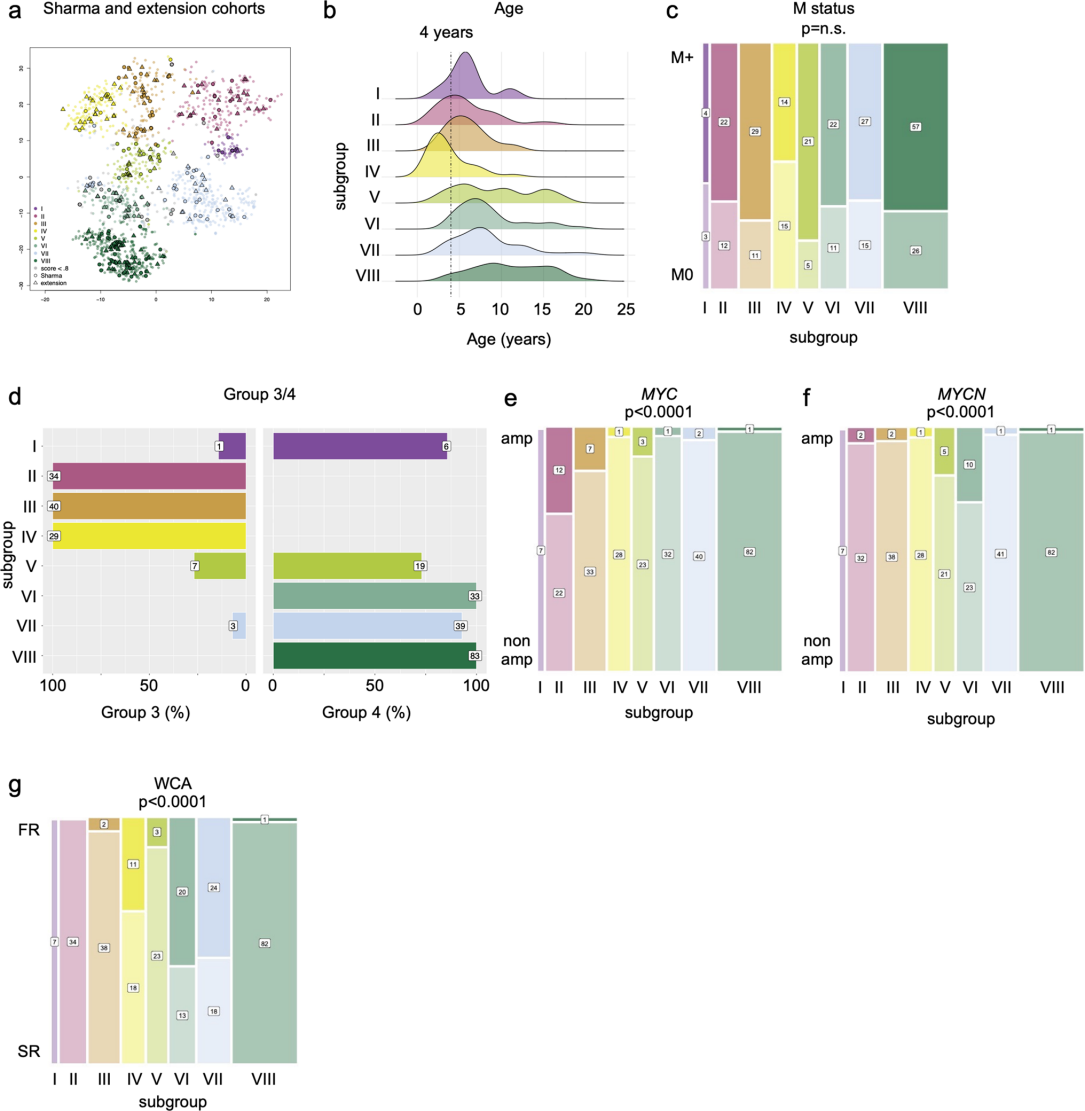
Methylation profiling of the study cohort. **a** t-sne plot of methylation profiles of samples from the HIT cohort (Sharma and extension) included in the study, in reference to the Group 3/4 subtypes consensus cohort [24]. **b** Ridge plot of age distribution across subgroups I–VIII. **c** Distribution of metastatic (M-) status across subgroups I–VIII. **d** Distribution of subgroups I–VIII across Group 3 or 4. Percentages indicate percent in each subgroup. **e** Distribution of MYC non-amplified (non amp) and amplified (amp) cases across subgroups, numbers indicate *n* cases. **f** Distribution of MYCN non-amplified (non amp) and amplified (amp) cases across subgroups, numbers indicate *n* cases. **g** Distribution of WCA phenotypes (FR: favorable risk, SR: standard risk) across subgroups, numbers indicate n cases

Three-hundred forty-six of 403 patients of the HIT cohort (*n* = 163 Sharma, *n* = 183 extension cohort) met the eligibility criteria (Fig. 1a). Concordance analysis of classifier versions v11b6 (used in this study) and the newer v12.5 for Group 3 or 4 classification, and classifier mb_g34 (used in this study) and the newer v12.5 for subgroup I–VIII classification revealed almost perfect agreements between the results of the respective classifier results (Fig. 1b). For clinical and molecular variables of the HIT cohort (Sharma and extension) see Table 1. 294/346 (73%) were included in further analyses based on Group 3 or 4 score (v11b6) ≥ 0.8 and subgroup I–VIII score (mb_g34) ≥ 0.8 (Figs. 1a and 2a).

**Table 1:** Clinical and molecular data (HIT cohort)

Characteristic	HIT Sharma cohort *n* = 147 [No. of patients (%)]	HIT extension cohort *n* = 147 [No. of patients (%)]	*p* value (Pearson’s *χ*^2^ test) (level of significance: 95%)
Age at diagnosis			
< 4 years	46 (31.3%)	12 (8.2%)	***0.00***
≥ 4 years	101 (68.7%)	135 (91.8%)	
Sex			
Male	112 (76.2%)	107 (72.8%)	0.5
Female	35 (23.8%)	40 (27.2%)	
Initial staging			
R0/M0	43 (29.3%)	43 (29.3%)	0.8
R + /M0	7 (4.8%)	5 (3.4%)	
M +	97 (65.9%)	99 (67.3%)	
Histology, centrally reviewed			
CMB	124 (84.3%)	135 (91.8%)	***0.01***
DMB	2 (1.4%)	5 (3.4%)	
LC/AMB	21 (14.3%)	7 (4.8%)	
Molecular group			
MB, Group 3	68 (46.3%)	46 (31.3%)	***0.01***
MB, Group 4	79 (53.7%)	101 (68.7%)	
Molecular subgroup			
I	1 (0.7%)	6 (4.1%)	***0.01***
II	21 (14.3%)	13 (8.8%)	
III	19 (12.9%)	21 (14.3%)	
IV	23 (15.6%)	6 (4.1%)	
V	13 (8.8%)	13 (8.8%)	
VI	16 (10.9%)	17 (11.6%)	
VII	16 (10.9%)	26 (17.7%)	
VIII	38 (25.9%)	45 (30.6%)	
Whole chromosomal aberrations			
≥ 2 (LR)	29 (19.7%)	32 (21.8%)	0.7
< 2 (SR)	118 (80.3%)	115 (78.2%)	
Iso-chromosome 17q			
Yes	98 (66.7%)	86 (58.5%)	0.1
No	49 (33.3%)	61 (41.5%)	
*MYC/N* amplification			
No amplification	117 (79.6%)	130 (88.4%)	0.2
* MYC*	15 (10.2%)	10 (6.8%)	
* MYCN*	14 (9.5%)	7 (4.8%)	
* MYC* and *MYCN*	1 (0.7%)	0	

*p* < 0.05 was considered significant (in bold italic)

Distribution of age across subgroups was similar as previously described (Fig. 2b). Subgroups III (29/40; 72.5%) and V (21/26; 80.8%) had the highest, and subgroup IV (14/29; 48.3%) the lowest proportion of M + cases (Fig. 2c). Both distribution across subgroups I–VIII as well as correlation of subgroups with Group 3 and 4 (Fig. 2d; Supplemental Fig. 3, online resource) followed pre-published principles [20, 24]. Subgroups II, III, and IV were exclusively Group 3, while subgroups I and V–VIII were mainly or exclusively Group 4 (Fig. 2d). *MYC* amplifications were predominantly seen in subgroups II and III (Fig. 2e; Supplemental Fig. 3, online resource), *MYCN* amplifications in subgroups V and VI (Fig. 2f; Supplemental Fig. 3, online resource). The majority of WCA favorable risk (FR) phenotype cases, characterized by presence of two or more of gain of chr7, or loss of chr8 or chr11, were subgroups IV, VI, and VII (Fig. 2g; Supplemental Fig. 3 and Supplemental Table 2, online resources).

### Survival analysis

**Fig. 3:**
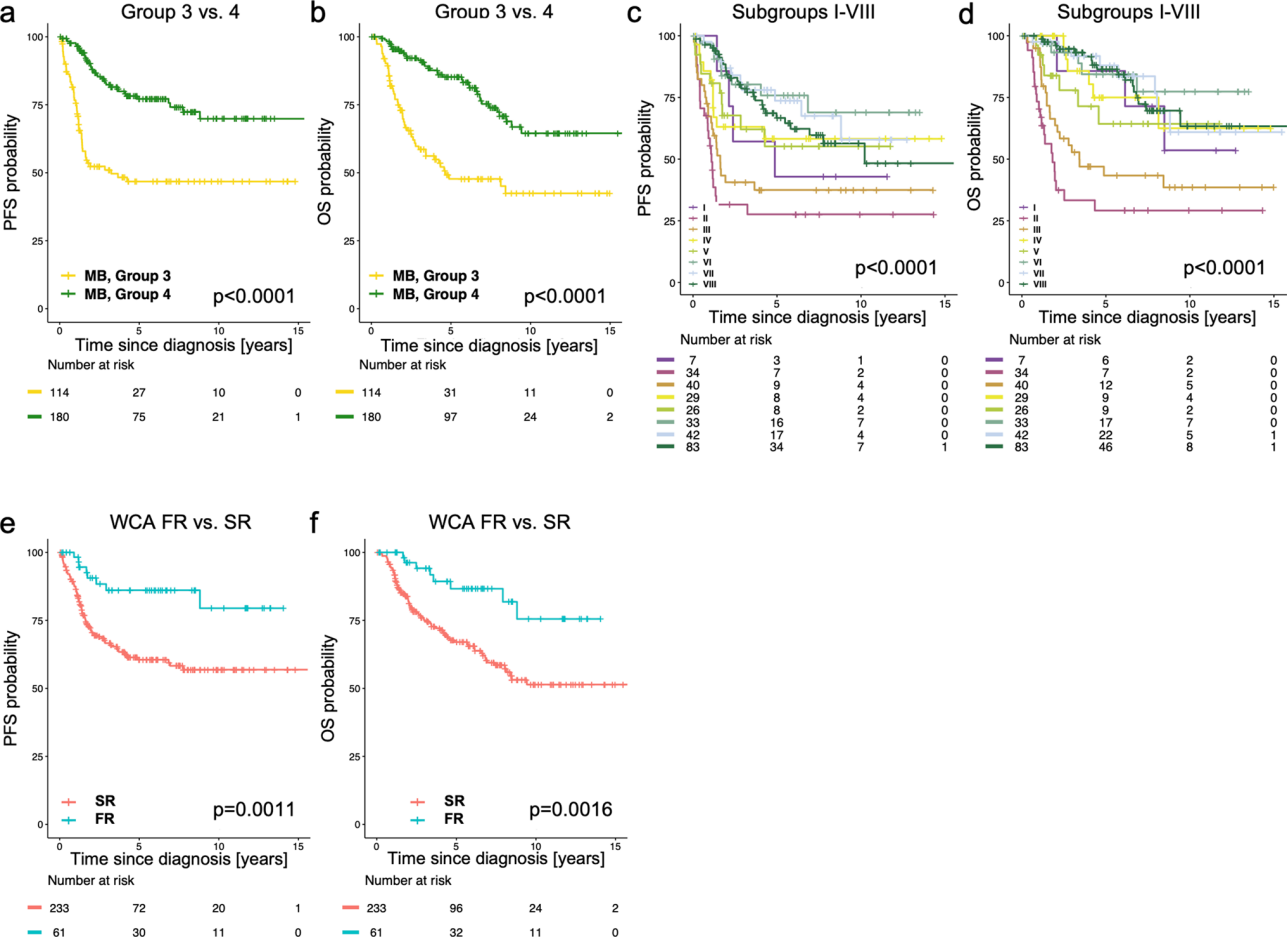
Survival analysis of the complete HIT cohort. **a** and **b** PFS and OS, comparison between Group 3 and Group 4 (HIT cohort). **c** and **d** PFS and OS, comparison between subtypes I–VIII (HIT cohort). **e** and **f** PFS and OS, comparison between WCA FR and SR (HIT cohort). Log rank testing, *p* < 0.05 was considered significant. *PFS* progression-free survival, *OS* overall survival, *WCA* whole-chromosomal aberrations, *FR* favorable risk, *SR* standard risk

PFS and OS were comparable in both the Sharma and the extension cohort (Supplemental Fig. 4, online resource) and showed only marginal differences in clinical parameters (Table 1). We thus combined both cohorts (HIT cohort) for further analysis. Patients with Group 3 or Group 4 MB differed significantly in both PFS and OS, with Group 3 MB showing a poorer outcome (5-year PFS: 41.4 ± 4.6%, 5-year OS: 48.8 ± 5.0%) compared to Group 4 MB (5-year PFS: 68.2 ± 3.7%, 5-year OS: 84.8 ± 2.8%) (Fig. 3a, b). Group 4 MB showed recurrences at later time points compared to Group 3 MB (Fig. 3a) with a significantly longer mean time to progression (Supplemental Table 3, online resource), as well as significantly longer time to death (Supplemental Table 3, online resource), while all recurrences and most deaths in Group 3 occurred within five years of diagnosis. Comparison by subgroup showed significant differences between subgroups, with subgroups II and III showing the poorest PFS (5-year PFS for II: 27.6 ± 8.2%, 5-year PFS for III: 37.5 ± 7.9%) and OS (5-year OS for II: 28.8 ± 8.7%, 5-year OS for III: 43.3 ± 8.3%), respectively (Fig. 3c, d). Subgroups VI, VII and VIII showed the best PFS (5-year PFS for VI: 76.6 ± 7.9%, 5-year PFS for VII: 75.9 ± 7.2%, 5-year PFS for VIII: 66.6 ± 5.8%; Fig. 3c, d). When separated by age, subgroup IV (all Group 3) in patients ≥ 4 years old was found to have an excellent outcome in the HIT cohort, with no events recorded (5-year PFS and OS: 100%) (Supplemental Fig. 5a, b, online resource). This however was not reproducible in the validation cohort, where no difference in PFS and OS between ≥ 4 and < 4 years of age was detected (Supplemental Fig. 5c, d, online resource). WCA FR showed significantly better PFS and OS compared to WCA SR (5-year PFS: 79.5 ± 5.6% vs 51.9 ± 3.6%, *p* < 0.001; 5-year OS: 86.6 ± 5.2% vs 67.0 ± 3.4%, *p* = 0.002), as expected (Fig. 3e, f). When SR only was analyzed, this trend was retained, but significance was not reached (PFS: *p* = 0.078, OS: *p* = 0.24; Supplemental Fig. 1 and 2, online resource). WCA-FR was inversely correlated with subgroup II or III (0/34 subgroup II and 2/40 subgroup III MBs belonged to the WCA-FR group), but highly correlated with subtype VII (7/10 clinical SR subgroup VII patients were WCA-FR).

### Multivariate survival analysis

**Fig. 4:**
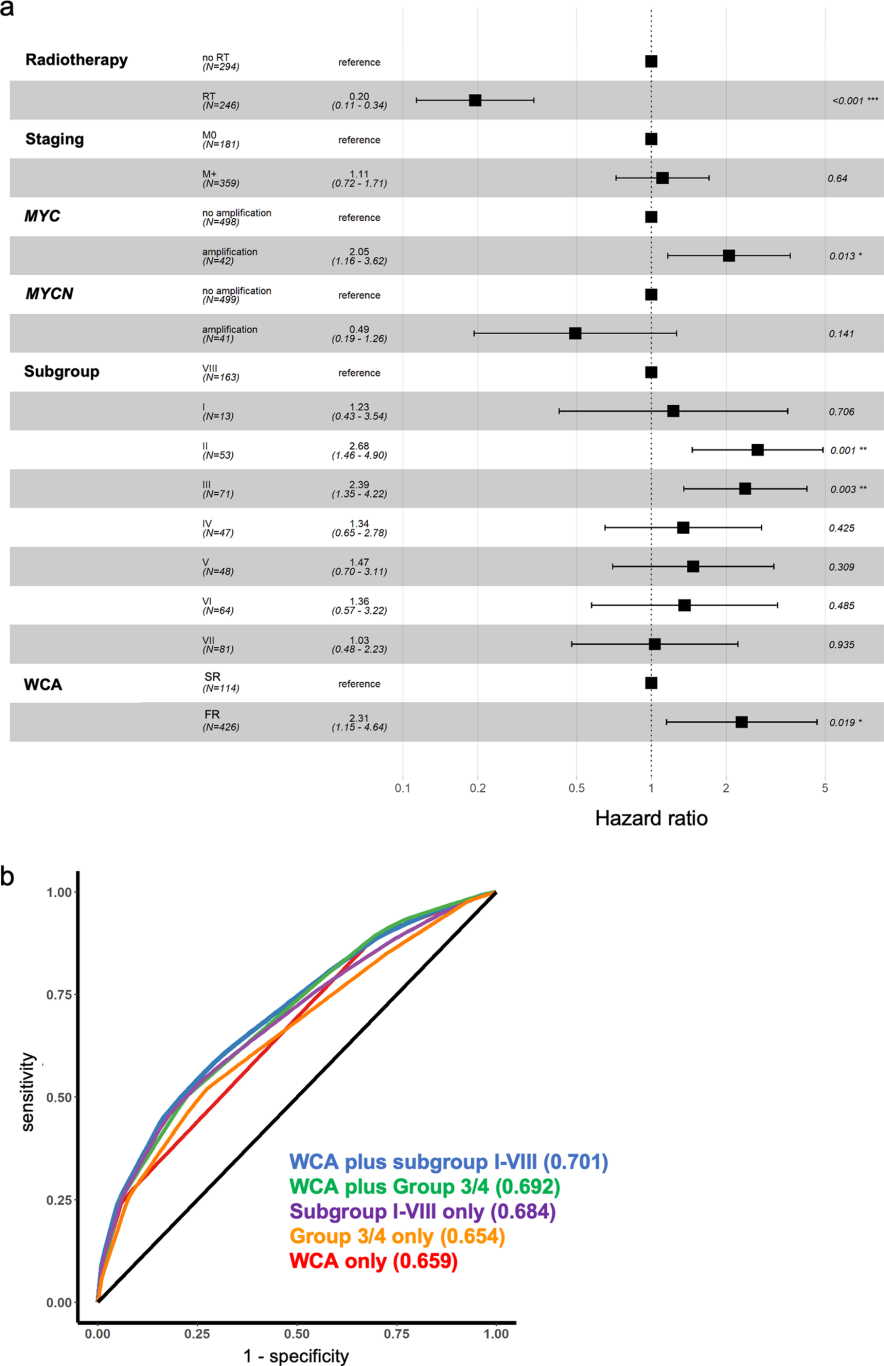
Risk factor analysis. **a** Hazard ratio Forest plot of multivariate Cox-regression analyses of risk factors for PFS in the HIT cohort. Radiotherapy (RT): time-dependent covariate. Number of events: 118. Global *p* value (Log Rank): 1.0764e–12; AIC: 1184.78; Concordance Index: 0.76. n.s.: not significant. **b** ROC-curve for 5-year-PFS in different Cox-regression models, including AUC values in parentheses. All models contained the standard clinical parameters radiotherapy as time-dependent covariate, staging, MYC-amplification and MYCN-amplification together with the mentioned biological parameters. Forest plots of the models underlying the ROC-curves can be found in supplemental Fig. 8 and 9 (online resource)

To quantify biological risk factors in a heterogeneously treated cohort, we used multivariate Cox-regression analysis. We established different models for the quantification of the effect of biological factors on progression-free survival, adjusting for use of radiotherapy (as time-dependent covariate), staging and presence of *MYC*/*MYCN* amplifications. On this backbone, we added either molecular group (Group 3/4) alone, subgroup (I–VIII) alone, WCA phenotype alone, WCA phenotype plus molecular group or WCA phenotype plus subgroup, and estimated the model fit of each of the models using the AUC of a ROC-analysis for 5-year PFS (Fig. 4b). Using this approach, we identified the combination of subgroup (I–VIII) with WCA as the model with the highest AUC (0.701), followed by the combination of molecular group (Group 3/4) with WCA (AUC 0.692) and subgroup (I–VIII) alone (AUC 0.684). This led us to select the combination of WCA and subgroup as the most powerful combination of biological parameters for further risk modeling.

### Risk stratification modeling

**Fig. 5:**
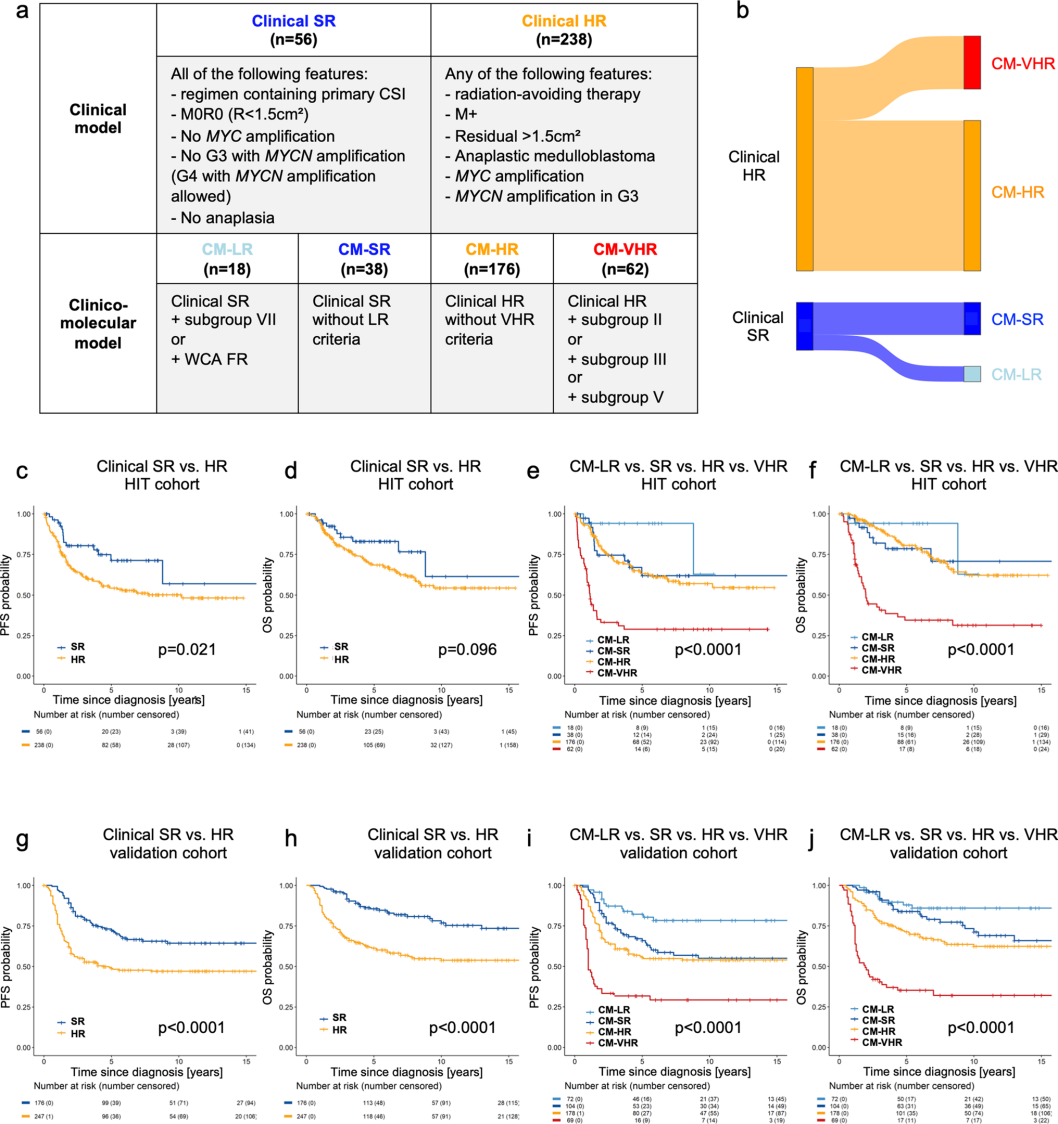
Risk factor modeling. **a** Proposed model including clinical and molecular parameters. **b** Sankey plot of cases allocated to strata of clinical model (left) and to strata of clinico-molecular model (right). **c** and **d** PFS and OS comparison between Clinical SR (clinical standard risk: regimen containing primary CSI, R0, and M0) and Clinical HR (clinical high risk: infant-type therapy regimen, R + , or M +) strata of the clinical model (HIT cohort). **e** and **f** PFS and OS, comparison between CM-LR, -SR, -HR and -VHR (clinico-molecular model) strata (HIT cohort). **g** and **h** PFS and OS comparison between Clinical SR (clinical standard risk: regimen containing primary CSI, R0, and M0) and Clinical HR (clinical high risk: infant-type therapy regimen, R + , or M +) strata of the clinical model (validation cohort). **i** and **j** PFS and OS, comparison between CM-LR, -SR, -HR and -VHR (clinico-molecular model) strata (validation cohort). Log rank testing, *p* < 0.05 was considered significant. *PFS* progression-free survival, *OS* overall survival, *LR* low risk, *SR* standard risk, *HR* high risk, *VHR* very high risk

Currently, risk-assessment and subsequent clinical decision making regarding therapy intensity are strongly based on clinical parameters, which are furthermore considered to be a function of the underlying biology. We therefore developed a model integrating clinical parameters with subgroups and WCA, reducing the potential number of combined molecular risk groups based on subgroup and WCA phenotype to a clinically applicable classification. Based on stratification schemes currently being evaluated in European trials such as SIOP PNET5, we defined clinically standard risk (clinical SR) patients as completely resected (*R* < 1.5 cm^2^), non-metastatic, non-anaplastic MB without *MYC* or *MYCN* (unless Group 4) amplification in a child older than 4 years at diagnosis. All other patients were stratified as clinically high-risk (clinical HR) (Fig. 5a). As suggested by the multivariate analysis, risk modeling solely based on clinical criteria did not predict survival in an ideal manner (Fig. 5c, d). For the development of an integrated clinico-molecular risk classification, we added the molecular risk factors subgroup II, III, V for very high risk (CM-VHR), and VII and WCA FR for low risk (CM-LR), based on published data [7, 9] and strong overlap of subgroup VII with the WCA-FR phenotype as described above (Fig. 5a, b), and additional analyses on the interplay between subgroup VII and WCA-FR (supplemental Fig. 6, online resource). Integration of these novel molecular risk factors with the clinical model to a “clinico-molecular” model (Fig. 5a, b) led to a highly informative risk modeling in non-WNT/non-SHH MB: both 5-year PFS and OS in the CM-LR stratum were 94 ± 5.7%, while in the CM-VHR stratum 5-year PFS was as low as 29 ± 6.1% and 5-year OS was 35 ± 6.5% (Fig. 5e, f). Upon bootstrapped cross-validation the new clinico-molecular model outperformed the clinical model in predicting PFS (clinical model: IBS 0.186/C-Index at 5-years 0.549 vs. clinico-molecular model: IBS 0.168/C-Index at 5 years 0.641, Supplemental Fig. 7, online resource).

Of note, both events in the CM-LR stratum were not relapses: one of these patients died of a pneumonia during chemotherapy and one further patient died of a second malignancy. It is of particular interest to note, that addition of information of subgroup VII/WCA-FR in the clinical SR stratum identified patients (CM-SR) clinically regarded as standard risk to have a poor prognosis almost identical to clinically high-risk patients without additional high-risk factors (CM-HR) (Fig. 5e, f).

For validation of the clinico-molecular model, this stratification was applied to a validation cohort of 423 non-WNT/non-SHH MB. For clinical and molecular variables of the validation cohort see Table 2. Compared to the HIT cohort, the validation cohort had a higher proportion of R + /M0, of LCA MB and of WCA FR cases (Table 2). No significant differences were detected for the other parameters, including PFS and OS. Again, risk modeling solely based on clinical criteria did not predict survival well with a 5-year PFS of 75.3 ± 4.7% (SR) vs. 55.3 ± 2.8% (HR) and a 5-year OS of 87.7 ± 3.9 (SR) vs. 67.2 ± 2.6% (HR) (Fig. 5g, h). However, similar to the HIT cohort, application of the clinico-molecular model led to an improved risk prediction: the CM-LR stratum of the validation cohort showed a favorable 5-year PFS and OS of 82.1 ± 6.0% and 90.5 ± 5.3% (Fig. 5i, j), respectively, and the CM-VHR stratum a very poor 5-year PFS and 5-year OS of 47.5 ± 4.1% and 55.0 ± 4.2%, respectively (Fig. 5j, h). The CM-SR and CM-HR strata again had very similar survival outcomes upon addition of the molecular parameters, despite being clinically regarded as standard and high risk, respectively. Confirming the results in the discovery cohort, the clinico-molecular model outperformed the clinical model in both the IBS (0.166 vs 0.180) and the C-index (0.667 vs 0.614), indicating superiority of the clinico-molecular model.

**Table 2:** Clinical and molecular data (validation cohort)

Distribution of clinical and molecular characteristics	HIT combined cohort *n* = 294 [No. of patients (%)]	International validation cohort *n* = 423 [No. of patients (%)]	*p* value (Pearson’s *χ*^2^ test) (level of significance: 95%)
Age at diagnosis			
< 4 years	58 (19.7%)	71 (16.8%)	0.3
≥ 4 years	236 (80.3%)	352 (83.2%)	
Sex			
Male	219 (74.5%)	295 (69.7%)	0.2
Female	75 (25.5%)	128 (30.3%)	
Initial staging			
R0/M0	86 (29.3%)	110 (26.0%)	***0.00***
R + /M0	12 (4.0%)	90 (21.3%)	
M +	196 (66.7%)	223 (52.7%)	
Histology			
CMB	259 (88.1%)	341 (80.6%)	***0.00***
DMB	7 (2.4%)	0	
LC/AMB	28 (9.5%)	82 (19.4%)	
Molecular group			
MB, Group 3	114 (38.8%)	165 (39.0%)	0.1
MB, Group 4	180 (61.2%)	258 (61.0%)	
Molecular subgroup			
I	7 (2.4%)	10 (2.4%)	0.2
II	34 (11.6%)	66 (15.6%)	
III	40 (13.6%)	35 (8.3%)	
IV	29 (9.9%)	44 (10.4%)	
V	26 (8.8%)	37 (8.8%)	
VI	33 (11.2%)	61 (14.4%)	
VII	42 (14.3%)	67 (15.8%)	
VIII	83 (28.2%)	103 (24.3%)	
Whole chromosomal aberrations			
≥ 2 (FR)	61 (20.8%)	143 (33.8%)	***0.00***
< 2 (SR)	233 (79.2%)	280 (66.2%)	
Iso-chromosome 17q			
Yes	184 (62.6%)	253 (59.8%)	0.5
No	110 (37.4%)	170 (40.2%)	
*MYC/N* amplification			
No amplification	247 (84.0%)	366 (86.5%)	0.5
* MYC*	25 (8.5%)	29 (6.9%)	
* MYCN*	21 (7.1%)	28 (6.6%)	
* MYC* and *MYCN*	1 (0.4%)	0	

Kaplan Meier survival estimation	HIT combined cohort *n* = 294	International validation cohort *n* = 423	*p* value (Log Rank) (level of significance: 95%)
5-year progression-free survival (PFS) (± standard deviation)	57.4 ± 3.2%	59.3 ± 2.4%	0.5
5-year overall survival (OS) (years; ± standard deviation)	71.0 ± 3.0%	71.3 ± 2.3%	0.5

*p* < 0.05 was considered significant (in bold italic)

In conclusion, addition of novel molecular risk markers such as methylation subgroups and WCA phenotype identifies prognostic strata clearly distinct from clinical risk categories.

## Discussion

DNA methylation-based classification of tumors is a very powerful tool to molecularly classify pediatric brain tumors and to infer the risk of relapse for the individual patient [3]. Several examples for the application of this technology have been described in pediatric MB [4, 20, 23, 24], but application in clinical decision making is not fully established yet. In addition, analyses available so far did not integrate DNA methylation-based classifications with information on whole-chromosomal aberrations, which identify patients with a low risk for relapse [9, 25].

In this analysis, we integrate two pre-defined classification schemes, second generation DNA methylation-based classification [24] (subgroups I–VIII) and whole-chromosomal aberration phenotypes [9] (WCA-FR and -SR), with standard clinical data to improve risk stratification in childhood non-WNT/non-SHH (Group 3/4) MB. While demographic and key survival data according to subgroup I-VIII in this cohort were compatible to previous reports [24], the pattern of the WCA phenotype outside a clinically standard-risk medulloblastoma population [9] was previously not described. Upon integration of clinical and molecular data, we were able to identify patients at very low risk for relapse, defined by presence of subgroup VII and/or WCA-FR phenotype in a clinical standard risk background (CM-LR). These patients, accounting for 6% of patients with non-WNT/non-SHH MB in the HIT cohort, have a risk of relapse comparable to the relapse risk of WNT-activated MB. Most importantly, we were able to validate this stratification scheme in an independent, published cohort of non-WNT/non-SHH MB (validation cohort), providing further evidence of valid discrimination in this MB group. Thus, the identification of non-WNT/non-SHH MB patients with a very favorable risk profile is possible, based on clinical and molecular factors.

On the other hand, patients with a clinical high-risk profile and subgroup II, III or V MB (CM-VHR, 21% of patients with non-WNT/non-SHH MB) have a chance as small as 30% to survive their disease without relapse, constituting a group of very high-risk patients. It is important to note, that DNA-methylation based subgrouping added significant information to clinical stratification. This is shown by our data, where patients with a clinical standard-risk profile without molecular low-risk profile (CM-SR) fare significantly worse than patients with clinical standard-risk MB with a molecular low-risk profile, i.e. subgroup VII and/or WCA-FR (CM-LR). Patients with a clinical standard-risk and biological high-risk markers (CM-SR) displayed an outcome comparable to clinical high-risk patients without biological high-risk markers (CM-HR). Based on their almost identical outcomes, it could therefore be considered to treat both of these groups, CM-SR and -HR, with the same strategy, despite having a differing clinical risk profile.

The risk stratification we suggest is useful, because it enables further risk classification of non-WNT/non-SHH MB that can be easily integrated into clinical routine. Moreover, it is feasible because all additional information required is available from DNA-methylation profiling, a platform which is increasingly established in routine diagnostics in many centers, and which is considered to be part of a mandatory pre-inclusion molecular diagnostic investigation for many European childhood brain tumor trials, like SIOP-PNET5-MB (NCT02066220) [19] or SIOP-HRMB (Eudra-CT No 2018-004250-17) [2]. The scheme we suggest for the identification of low-risk non-WNT/non-SHH MB patients integrates and is in line with two published schemes to identify low-risk non-WNT/non-SHH Group 3/4 MB from the SIOP-PNET4 research group [9] and from the SJMB03 trial [7], respectively. Indeed, our data suggest that these two classifications may largely overlap, with the larger proportion of subgroup VII patients belonging to the WCA-FR subgroup, and many WCA-FR patients being subgroup VII. While the addition of WCA phenotypes to the subgroups adds prognostic information in our analysis, independency of the risk factors and additional benefit provided by the integration of both factors needs to be prospectively validated.

Our analysis has several limitations. The first and probably most important limitation is the relatively small proportion of standard risk patients included in this analysis, hereby weakening the ability to identify FR markers in this cohort. Data from PNET5 trial participants were not included in this series, limiting the number of clinically standard risk patients in the study cohort. This opens the option to further validate the findings of this study in a large and independent cohort, once the data from e.g. the SIOP PNET5 trial are available for analysis. Secondly, the selection into this study has been made based on availability of a DNA-methylation profiling results, instead of prospectively and systematically profiling a pre-defined cohort. While most of the data from DNA-methylation profiling is derived from prospective clinical trials or molecular diagnostic studies, it is possible that, especially in the case of diagnostic studies, centers choose to preferentially include patients considered to be at higher risk of relapse or only after relapse. However, we were able to validate our findings in an independent MB cohort, substantiating our clinico-molecular stratification scheme. Furthermore, in our study we have inferred CNV from methylation profiles, which may need validation for practical purposes. In e.g. polyploid cases it may be difficult to determine a diploid baseline from methylation profiles. This is in particular crucial for therapeutically relevant stratification markers such as WCA phenotype aberrations or amplifications of e.g. *MYC*, which needs to be validated by a second method within future clinical trials. Indeed, sensitivity of DNA-methylation array-based detection of MYC/MYCN amplifications might be lower than observed in our study [5]. iFISH is still the gold standard and should be used as the primary method for the detection of these amplifications in prospective trials. In this respect, unequivocal methylation subgrouping alone (i.e. without inclusion of copy number alterations) may pose an advantage regarding clean molecular stratification in a trial. However, stratification for patients with tumors failing methylation classification (in our study: 109/403; 27%) needs to be considered in such a setting.

One practical limitation of the PFS analysis was the definition of event we used in this study: patients were considered to have an event for the PFS analysis if they died from any cause. This was critical in the low-risk cohort CM-LR, where two events observed in this stratum were deaths unrelated to relapse (one second malignancy and one death due to pneumonia). These patients therefore cannot serve as arguments to further increase the intensity of therapy, and potentially might have even benefited from less intensive therapy to avoid complications. Finally, despite a large overall number of cases in this study, the case numbers within each subgroup become small when accounting for the eight subgroups of non-WNT/non-SHH MB, in particular when accounting for additional risk factors such as e.g. age (see subgroup IV). Thus, conclusions for practical clinical use need to be carefully considered, and should foremost influence future clinical trial design to enable prospective validation.

Based on the growing evidence for the possibility to further sub-classify non-WNT/non-SHH MB into different risk strata, the time has come to use these data to explore the potential for improved risk-adapted therapy. For the low-risk non-WNT/non-SHH CM-LR stratum, reduction of therapy intensity needs to be discussed in analogy to currently ongoing trials for WNT-activated MB, where children receive only 18 Gy CSI or even less (NCT01878617, NCT02724579, and NCT02066220). It is imperative that this decision must be made carefully and in the context of a clinical trial, as safety of intensity reduction in this particular cohort has not been shown so far. ACNS0331 reported inferior progression-free survival in younger children with clinically standard risk MB treated with reduced CSI dose. However, molecular characterization was not taken into account for therapy stratification [16], limiting the analysis based on molecular groups or subgroups. Since the same trial reported improved neurocognitive outcomes in patients treated with reduced CSI, the aim to reduce the CSI dose in patients where this is safe remains a high priority.

For the CM-SR stratum of patients with clinically standard risk non-WNT/non-SHH MB without biological low-risk markers, there is further need to improve outcomes given the 65% 5-year PFS with current therapy. We provide a scheme allowing identification of these CM-SR patients, which need to be treated with strategies of similar intensity as clinically high-risk patients. Again, an improved treatment strategy for this cohort of CM-SR and CM-HR patients remains to be carefully developed, considering both acute toxicity of intensified chemotherapy and the long-term neurotoxicity of increased radiotherapy dose. Finally, CM-VHR patients with very high-risk MB need very special consideration. With current standard protocols, prognosis remains dismal in these patients. Upon availability of promising experimental approaches, these patients should be discussed to be eligible for early phase trials during first-line treatment.

In summary, integration of clinical and molecular risk factors allows the identification of distinct low-, standard- and high-risk patients among non-WNT/non-SHH MB. Molecular factors add significant information to risk stratification for clinical use, and identify clinically low-risk patients with a high risk of relapse, eligible for intensified treatment. Group 3/4 subgroup VII and WCA phenotype, as well as subgroups II, III, and V, need to be considered for molecular stratification in future clinical trials. Additional cohorts such as patients ≥ 4 years with subgroup IV MB may need further investigation.

## Supplementary Information

Below is the link to the electronic supplementary material.

Supplementary file1 Supplemental Fig. 1: Survival analyses of progression-free survival (PFS) according to different combinations of whole-chromosomal aberration (WCA) markers in clinically standard risk patients. a single markers, b combination of two markers, c combination of all three markers. Log rank testing, p<0.05 was considered significant. PFS: progression-free survival; Chr: chromosome. Supplemental Fig. 2: Survival analyses of overall survival (OS) according to different combinations of whole-chromosomal aberration (WCA) markers in clinically standard risk patients. a single markers, b combination of two markers, c combination of all three markers. Log rank testing, p<0.05 was considered significant. OS: overall survival; Chr: chromosome. Supplemental Fig. 3: Clinico-molecular description of the study cohort. Distribution of clinical (age, M- and resection (R-) status) and molecular (WCA, MYCN, MYC, i17q, subtype I-VIII, CNVs) characteristics in the cohort ordered by subtypes. WCA: whole chromosome aberrations; chr: chromosome. Supplemental Fig. 4: Survival analysis. a and b PFS and OS, comparison between Sharma and extension cohorts. Log rank testing, p<0.05 was considered significant. PFS: progression-free survival; OS: overall survival. Supplemental Fig. 5: Survival analysis for subgroup IV. a and b: PFS and OS, only subgroup IV, comparison between age groups ≥/< 4years (HIT cohort). c and d: PFS and OS, only subgroup IV, comparison between age groups < 4 vs. ≥4 years (validation cohort). Log rank testing, p<0.05 was considered significant. PFS: progression-free survival; OS: overall survival. Supplemental Fig. 6: Survival analyses according to different potential good prognostic markers subgroup VII alone vs. WCA alone vs combination of subgroup VII and WCA FR in clinically standard risk patients. a PFS and OS, only clinically standard risk patients, comparison between subgroup VII vs. non-VII. b PFS and OS, only clinically standard risk patients, comparison between WCA FR vs. SR. c PFS and OS, only clinically standard risk patients, comparison between subgroup VII and/or WCA (low-risk, LR) vs. non-VII and WCA SR (standard risk, SR). Among the two events observed in the LR stratum, no event was due to relapse: one patient died of a second malignancy and the other died from a toxicity. Log rank testing, p<0.05 was considered significant. PFS: progression-free survival; OS: overall survival. Supplemental Fig. 7: Comparison of quality parameters of clinico-molecular vs. the clinical model. a prediction error curve comparing the clinico-molecular model with the clinical model, from main Fig. 6. b ROC-curve analysis comparing the clinico-molecular model vs. the clinical model in the discovery cohort, from main Fig. 6. Supplemental Fig. 8: Forest plots of Cox-regression analyses of alternative models for progression-free survival. a Cox regression with clinical model plus methylation subgroups I-VIII only. Number of events: 118. Global p value (Log Rank): 5.6286e-12; AIC: 1188.85; Concordance Index: 0.74. b Cox regression with clinical model plus methylation groups Group 3 and 4 only. Number of events: 118. Global p value (Log Rank): 1.55e-12; AIC: 1189.52; Concordance Index: 0.72. Supplemental Fig. 9: Forest plots of Cox-regression analyses of alternative models for progression-free survival. a Cox regression with clinical model plus WCA phenotype only. Number of events: 118. Global p value (Log Rank): 2.1526e-13; AIC: 1185 39; Concordance Index: 0 69. b Cox regression with clinical model plus methylation groups Group 3 and 4 plus WCA phenotype. Number of events: 118. Global p value (Log Rank): 1.3727e-14; AIC: 1178.67; Concordance Index: 0.74. Supplemental Table 1 Method comparison of MYC/MYCN-amplification detection by methylation profiling-derived copy number analysis, fluorescence in-situ hybridization (FISH), Molecular Inversion Profiling (MIP) or Multiplex Ligation-dependent Probe Amplification (MLPA). Supplemental Table 2 Clinical and molecular data of WCA-FR vs. -SR patients (HIT cohort), p<0.05 was considered significant. Supplemental Table 3 Mean time to event for Group 3 and Group 4 (HIT cohort). p<0.05 was considered significant. SD: standard deviation; min: minimum; max: maximum (PDF 6749 KB)

## Data Availability

The clinical and molecular data will be made available upon reasonable request to the corresponding authors in a GDPR conform manner.
