# Supplementary material for: Identification of low and very high-risk patients with non-WNT/non-SHH medulloblastoma by improved clinico-molecular stratification of the HIT2000 and I-HIT-MED cohorts
Source: Acta Neuropathol. 2022 Dec 2;145(1):97–112. doi: 10.1007/s00401-022-02522-4 (PMC9807480; doi:10.1007/s00401-022-02522-4)
Supplement: Supplementary file 1 — Supplementary file1 Supplemental Fig. 1: Survival analyses of progression-free survival (PFS) according to different combinations of whole-chromosomal aberration (WCA) markers in clinically standard risk patients. a single markers, b combination of two markers, c combination of all three markers. Log rank testing, p<0.05 was considered significant. PFS: progression-free survival; Chr: chromosome. Supplemental Fig. 2: Survival analyses of overall survival (OS) according to different combinations of whole-chromosomal aberration (WCA) markers in clinically standard risk patients. a single markers, b combination of two markers, c combination of all three markers. Log rank testing, p<0.05 was considered significant. OS: overall survival; Chr: chromosome. Supplemental Fig. 3: Clinico-molecular description of the study cohort. Distribution of clinical (age, M- and resection (R-) status) and molecular (WCA, MYCN, MYC, i17q, subtype I-VIII, CNVs) characteristics in the cohort ordered by subtypes. WCA: whole chromosome aberrations; chr: chromosome. Supplemental Fig. 4: Survival analysis. a and b PFS and OS, comparison between Sharma and extension cohorts. Log rank testing, p<0.05 was considered significant. PFS: progression-free survival; OS: overall survival. Supplemental Fig. 5: Survival analysis for subgroup IV. a and b: PFS and OS, only subgroup IV, comparison between age groups ≥/< 4years (HIT cohort). c and d: PFS and OS, only subgroup IV, comparison between age groups < 4 vs. ≥4 years (validation cohort). Log rank testing, p<0.05 was considered significant. PFS: progression-free survival; OS: overall survival. Supplemental Fig. 6: Survival analyses according to different potential good prognostic markers subgroup VII alone vs. WCA alone vs combination of subgroup VII and WCA FR in clinically standard risk patients. a PFS and OS, only clinically standard risk patients, comparison between subgroup VII vs. non-VII. b PFS and OS, only clinically standard risk patients, com [file 401_2022_2522_MOESM1_ESM.pdf]

Mynarek M et al., Molecular risk profiling in non-WNT/non-SHH medulloblastoma

Supplemental Figure 1

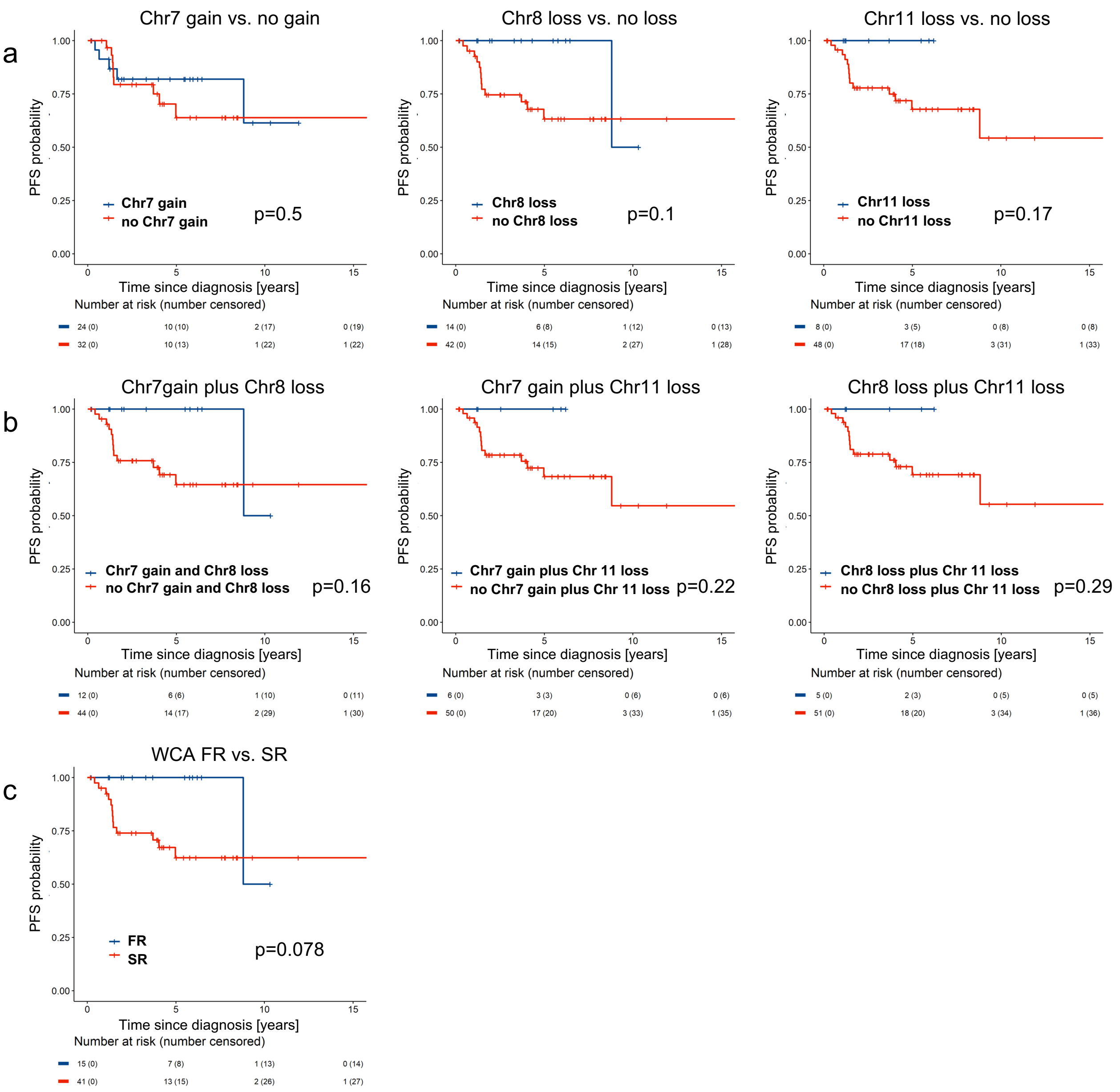

Mynarek M et al., Molecular risk profiling in non-WNT/non-SHH medulloblastoma

Supplemental Figure 2

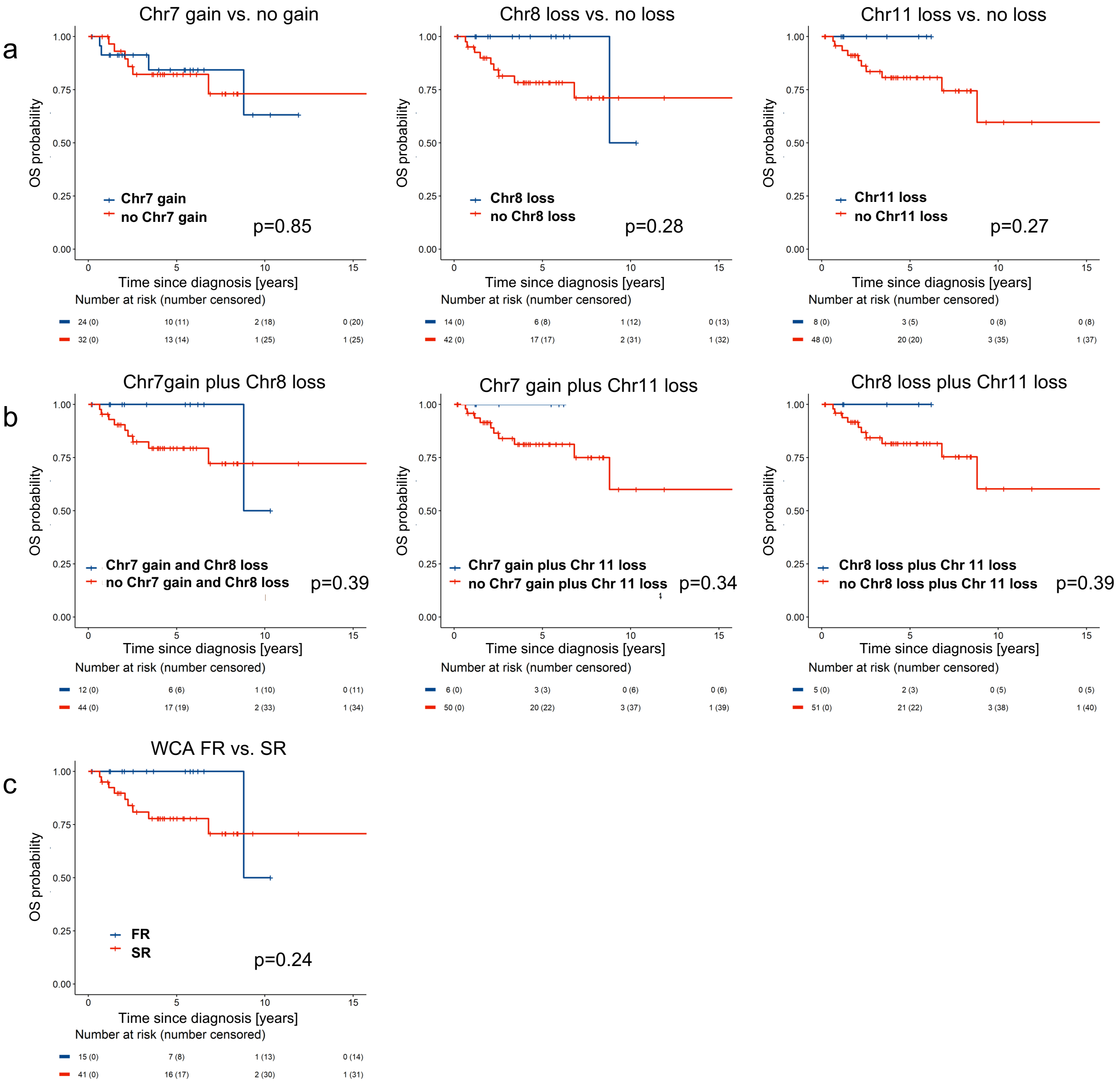

Mynarek M et al., Molecular risk profiling in non-WNT/non-SHH medulloblastoma

Supplemental Figure 3

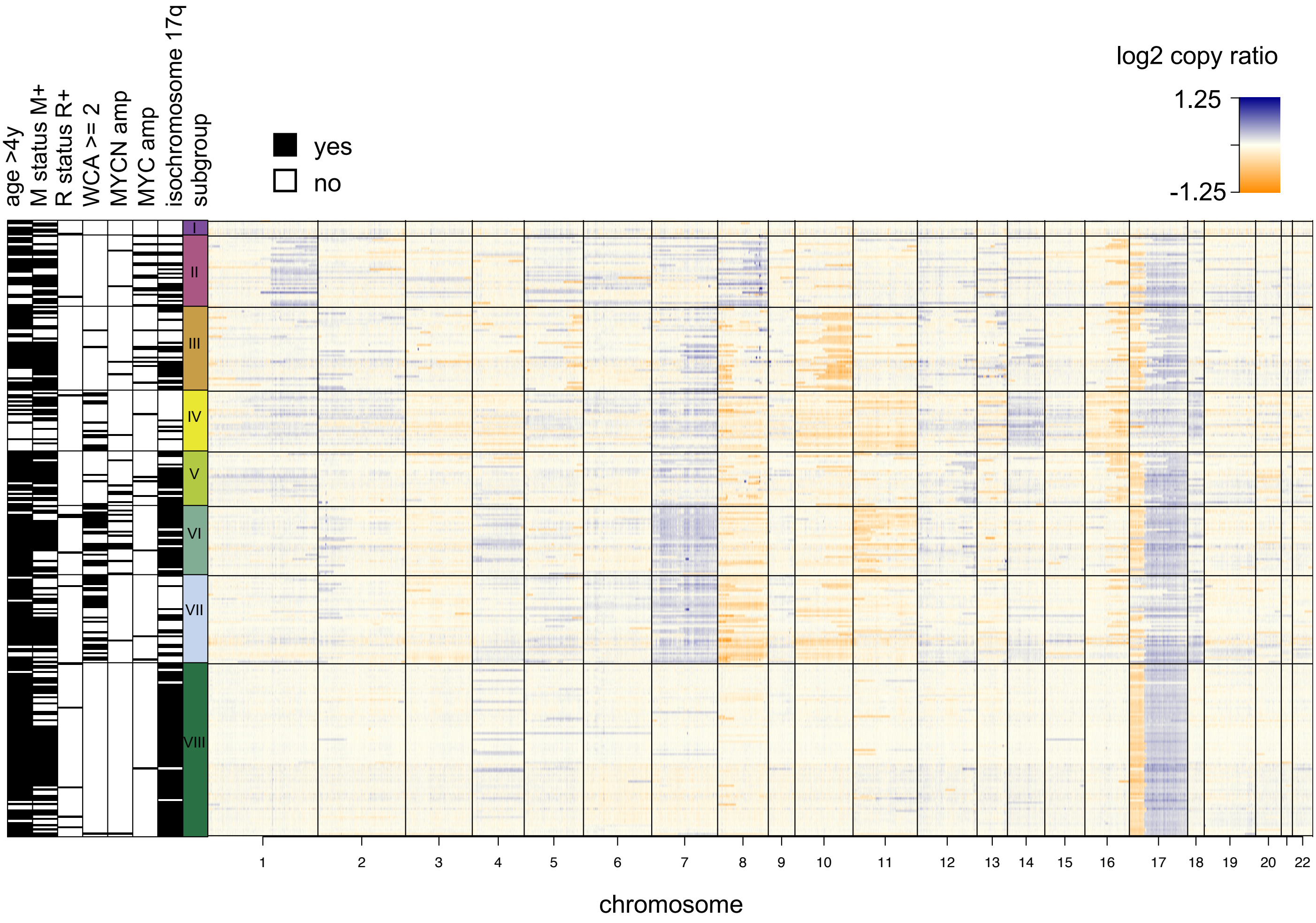

Mynarek M et al., Molecular risk profiling in non-WNT/non-SHH medulloblastoma

Supplemental Figure 4

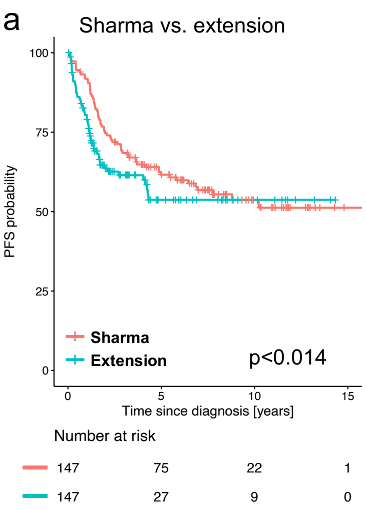

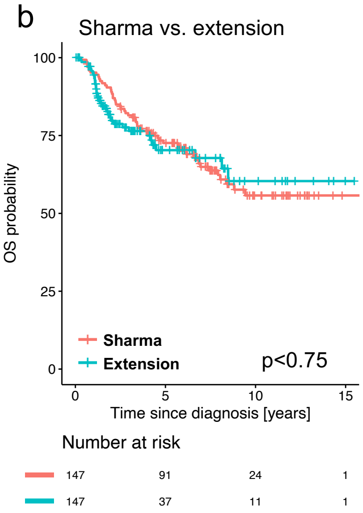

Mynarek M et al., Molecular risk profiling in non-WNT/non-SHH medulloblastoma

Supplemental Figure 5

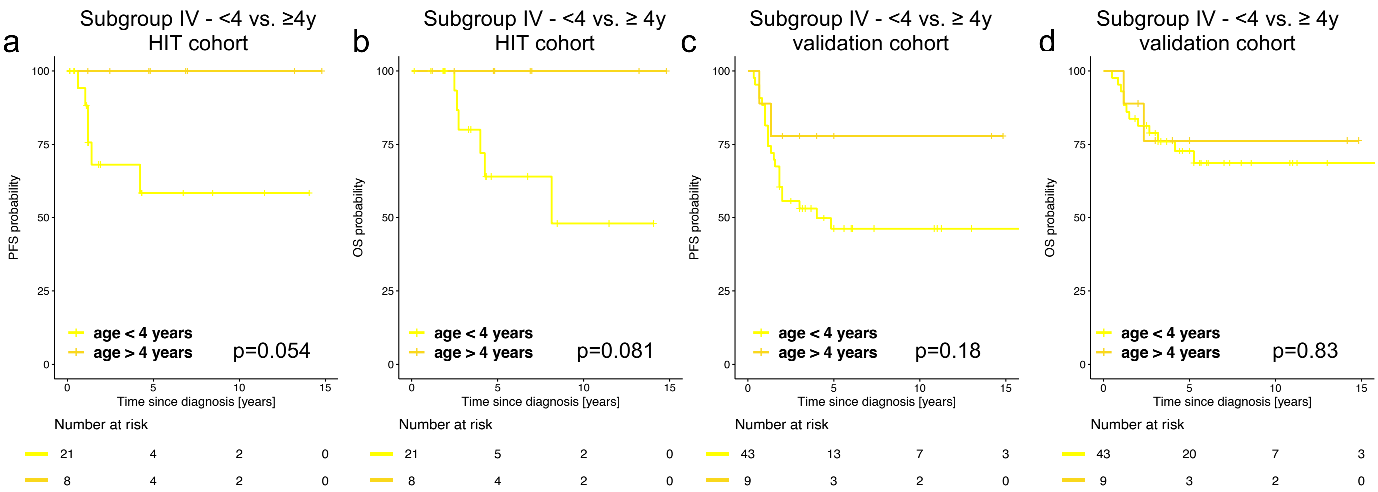

Mynarek M et al., Molecular risk profiling in non-WNT/non-SHH medulloblastoma

Supplemental Figure 6

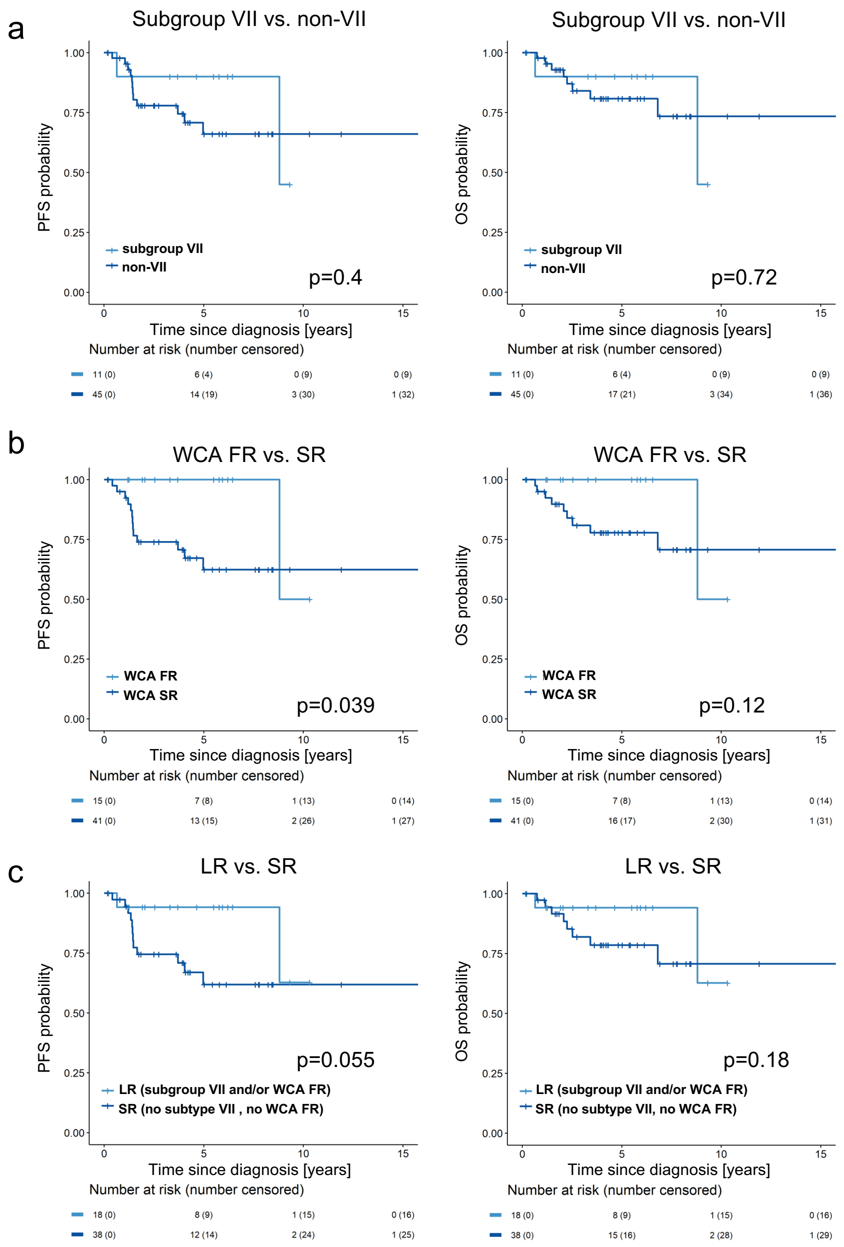

Mynarek M et al., Molecular risk profiling in non-WNT/non-SHH medulloblastoma

Supplemental Figure 7

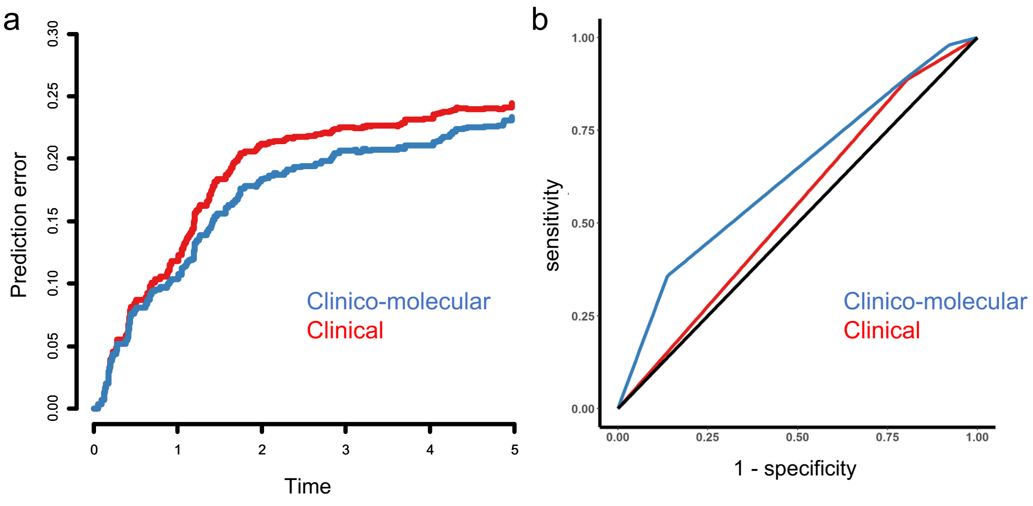

Mynarek M et al., Molecular risk profiling in non-WNT/non-SHH medulloblastoma

Supplemental Figure 8

a

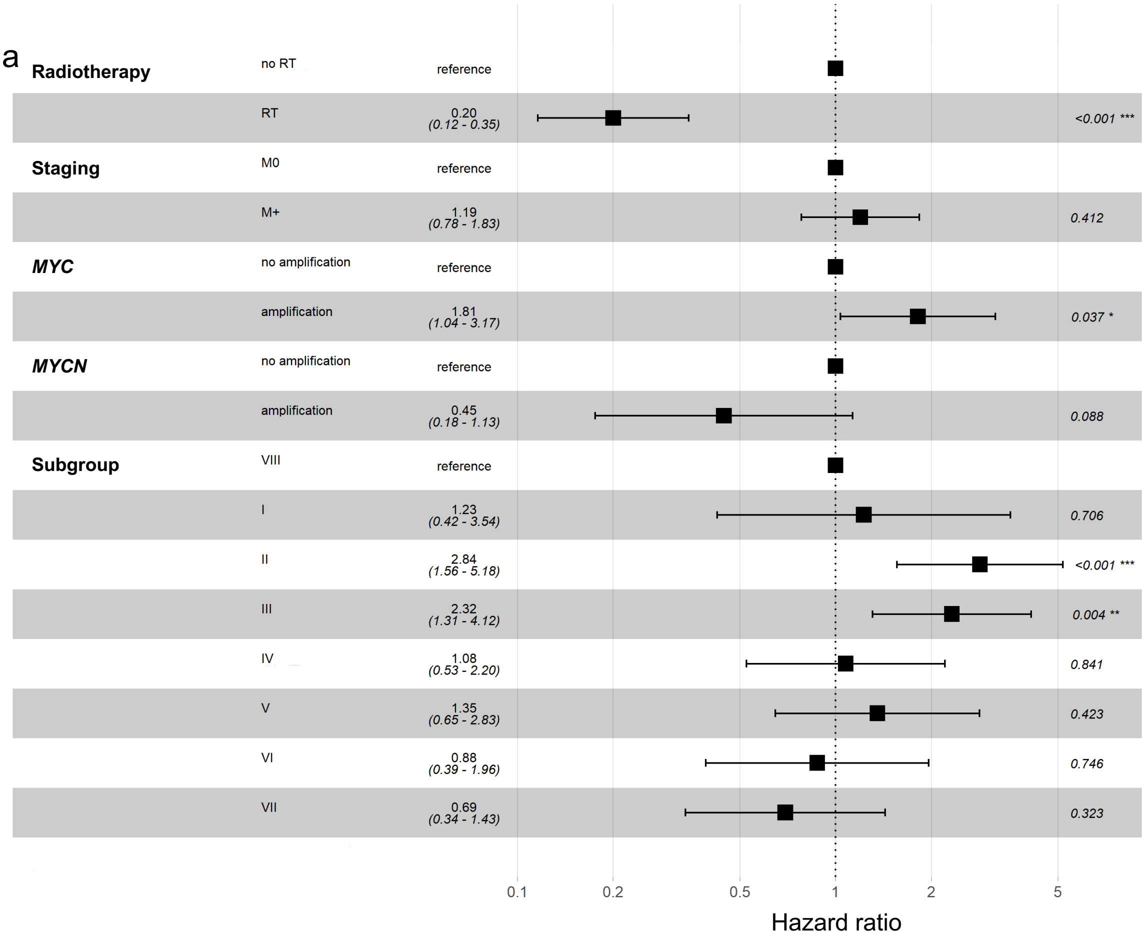

b

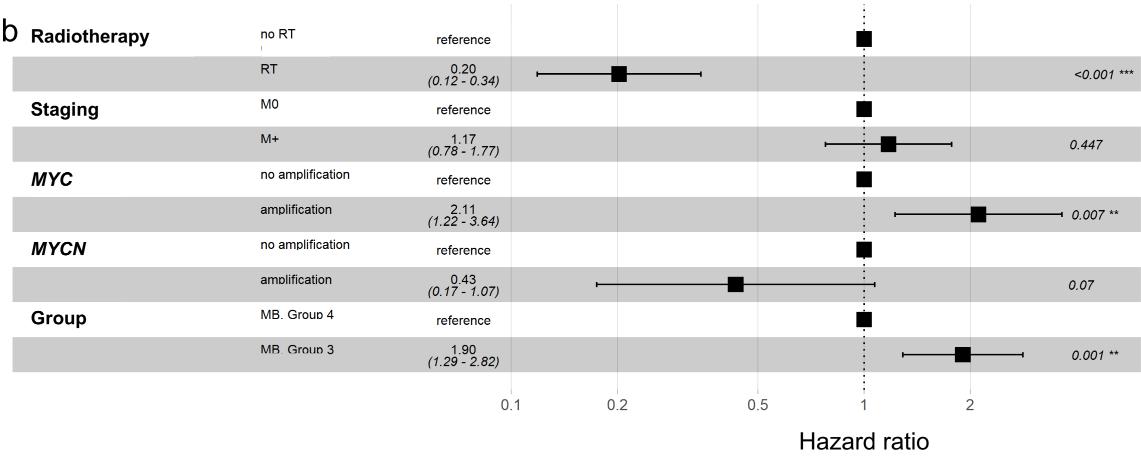

Mynarek M et al., Molecular risk profiling in non-WNT/non-SHH medulloblastoma

Supplemental Figure 9

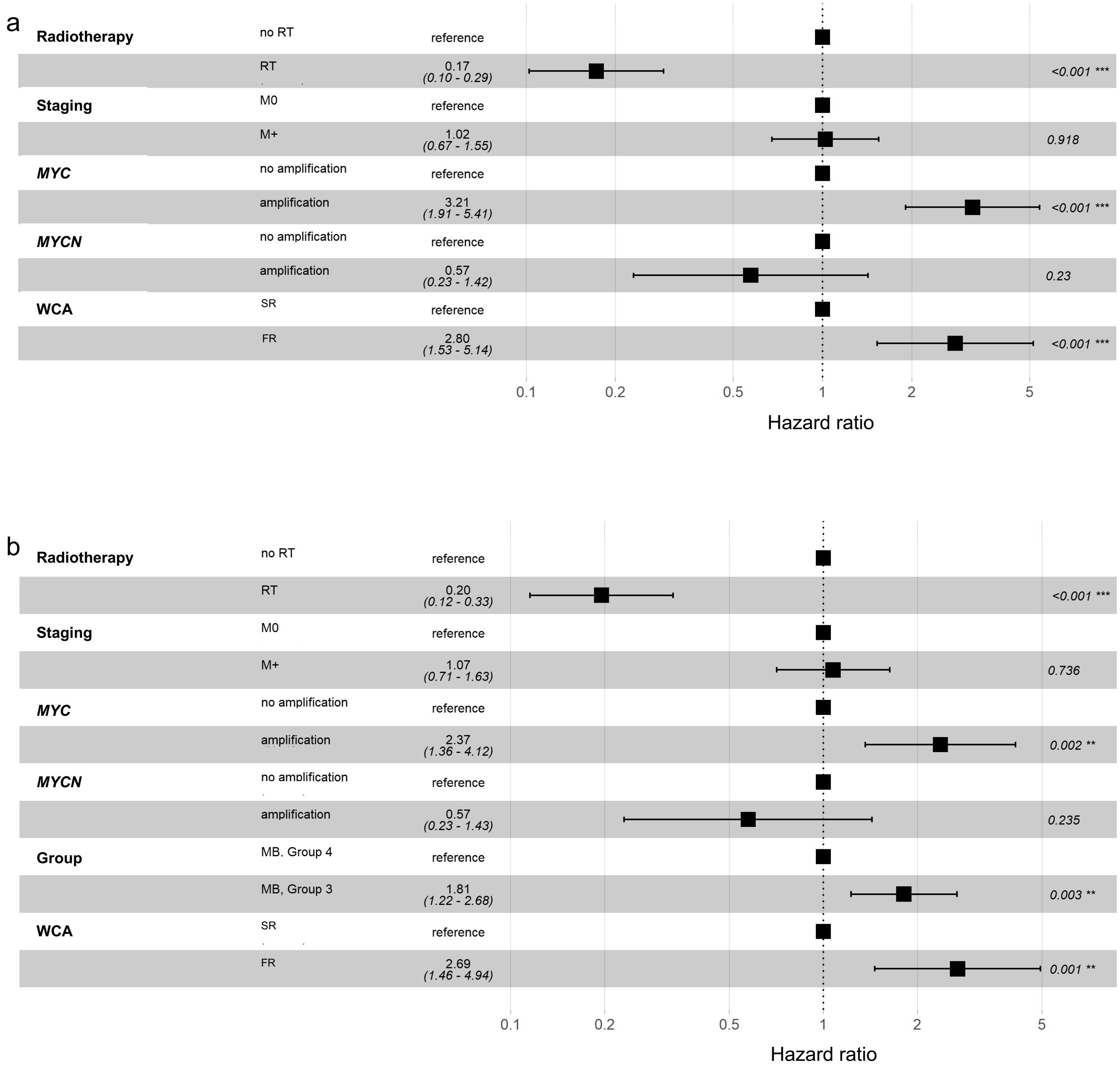

Supplementary Table 1:

***MYC* / *MYCN* Status by other method**		***MYC* / *MYCN* status by DNA methylation profiling**			**Total**
		**no amplification**	***MYC* amplification**	***MYCN* amplification**	
**FISH**	*MYC* amplified	0	2	0	2
	MYCN amplified	0	0	4	4
	no amplification	34	2	1	37
**MLPA**	*MYC* amplified	0	2		2
	no amplification	22	1		23
**MIP**	*MYC* amplified	0	9	0	9
	MYCN amplified	0	0	2	2
	no amplification	50	0	1	51
**Summary of FISH, MLPA and MIP**	***MYC* amplified**	**0**	**13**	**0**	**13**
	***MYCN* amplified**	**0**	**0**	**6**	**6**
	**no amplification**	**106**	**3**	**2**	**111**
**Total**		106	16	8	130

Concordance between methylation profiling-derived *MYC* / *MYCN* copy number analysis and FISH, MLPA or MIP: 0.88 [MYC] and 0.84 [MYCN] (Cohen's Kappa).

Mynarek M et al., Molecular risk profiling in non-WNT/non-SHH medulloblastoma

Supplementary Table 2:

**Characteristic**	**WCA FR**<br>**n=61**<br><br>[No. of patients (%)]	**WCA SR**<br>**n=233**<br><br>[No. of patients (%)]	**p-value**<br>**(Pearson's Chi-squared test)**<br>[level of significance: 95%]
**Staging**			
M0	27 (44%)	71 (30%)	0.060
M+	34 (56%)	162 (70%)	
**Age at diagnosis**			
<4 years	14 (23%)	44 (19%)	0.6
>4 years	47 (77%)	189 (81%)	
**Molecular subgroup**			
VIII	1 (1.6%)	82 (35%)	***<0.001***
I	0 (0%)	7 (3.0%)	
II	0 (0%)	34 (15%)	
III	2 (3.3%)	38 (16%)	
IV	11 (18%)	18 (7.7%)	
V	3 (4.9%)	23 (9.9%)	
VI	20 (33%)	13 (5.6%)	
VII	24 (39%)	18 (7.7%)	
**Molecular group**			
MB, Group 4	45 (74%)	135 (58%)	***0.035***
MB, Group 3	16 (26%)	98 (42%)	
**Histology**			
CMB/DMB	59 (97%)	207 (89%)	0.10
LCA	2 (3.3%)	26 (11%)	
MYCN **amplification**			
no amplification	51 (84%)	221 (95%)	***0.007***
amplification	10 (16%)	12 (5.2%)	
MYC **amplification**			
no amplification	56 (92%)	211 (91%)	>0.9
amplification	5 (8.2%)	22 (9.4%)	

Mynarek M et al., Molecular risk profiling in non-WNT/non-SHH medulloblastoma

Supplementary Table 3:

	**Mean time to progression ± SD (min-max) in years**	**Mean time to death ± SD (min-max) in years**	**Mean time from relapse to death ± SD (min-max) in years**
**MB Group 3**	0.98 ± 8.4 (0.05-4.2)	2.1 ± 1.7 (0.3-8.4)	1.0 ± 1.4 (0-7.5)
**MB Group 4**	2.1 ± 1.9 (0.1 – 10.2)	4.4 ± 2.6 (0.8-4.6)	1.6 ± 1.4 (0-6.3)
**Significance of difference (t-test)**	***<0.001***	***<0.001***	***0.07***
